# Mechanistic action of weak acid drugs on biofilms

**DOI:** 10.1038/s41598-017-05178-3

**Published:** 2017-07-06

**Authors:** Binu Kundukad, Megan Schussman, Kaiyuan Yang, Thomas Seviour, Liang Yang, Scott A. Rice, Staffan Kjelleberg, Patrick S. Doyle

**Affiliations:** 1 BioSystems and Micromechanics (BioSyM) IRG, Singapore MIT Alliance for Research and Technology (SMART), Singapore, Singapore; 2 Department of Brain and Cognitive Science, Massachusetts Institute of Technology, Cambridge, Massachusetts 02139 USA; 3 Department of Pharmacy, National University of Singapore, Singapore, Singapore; 4 Singapore Centre for Environmental Life Sciences Engineering, Nanyang Technological University, Singapore, Singapore; 5 School of Biological Sciences, Nanyang Technological University, Singapore, Singapore; 6 Centre for Marine Bio-Innovation and School of Biotechnology and Biomolecular Science, University of New South Wales, Sydney, NSW Australia; 7 Department of Chemical Engineering, Massachusetts Institute of Technology, Cambridge, Massachusetts 02139 USA

## Abstract

Selective permeability of a biofilm matrix to some drugs has resulted in the development of drug tolerant bacteria. Here we studied the efficacy of a weak organic acid drug, N-acetyl-L-cysteine (NAC), on the eradication of biofilms formed by the mucoid strain of *Pseudomonas aeruginosa* and investigated the commonality of this drug with that of acetic acid. We showed that NAC and acetic acid at pH < pKa can penetrate the matrix and eventually kill 100% of the bacteria embedded in the biofilm. Once the bacteria are killed, the microcolonies swell in size and passively shed bacteria, suggesting that the bacteria act as crosslinkers within the extracellular matrix. Despite shedding of the bacteria, the remnant matrix remains intact and behaves as a pH-responsive hydrogel. These studies not only have implications for drug design but also offer a route to generate robust soft matter materials.

## Introduction

Biofilms are colonies of bacteria held together by a self-secreted extracellular polymeric substance (EPS) called the matrix. The matrix protects the bacteria from adverse environmental conditions^1^ and hence poses a problem in the eradication of biofilms in clinical and environmental settings. *Pseudomonas aeruginosa* is of special interest because it is an opportunist pathogen responsible for infections in patients under a variety of conditions including burn wounds and indwelling medical devices^2, 3^. *P. aeruginosa* also infects the lungs of patients with cystic fibrosis and is known to undergo a switch to a mucoid phenotype that is characterized by the overproduction of alginate^4^. The EPS of *P. aeruginosa* is composed of a crosslinked network of polysaccharides, nucleic acids, proteins and other macromolecules that facilitate biofilm formation and maintenance^5^. Mucoid *P. aeruginosa* is known to form distinct microcolonies, which has been attributed to the presence of alginate in the matrix^5^. Mechanical property measurements have shown that the microcolonies have larger amounts of EPS as compared to the plains of the biofilm^6^. Microrheology studies have identified the contribution of different polysaccharides, namely Psl and Pel, on the viscoelastic properties of biofilm^7^. Overall, these studies have shown that the biofilm matrix is generally elastic and robust, though the elastic modulus varies depending on the type of bacteria, expression of polysaccharides, thickness of the biofilm and maturity of the biofilm^8–10^.

The presence of bacterial cells, the EPS, and other organic matter slows down diffusion in biofilms. The diffusive permeability has been found to decrease with increasing biomass volume fraction which suggests a serial resistance model of diffusion in biofilms^11, 12^. Though solute molecules as big as antibiotics have a diffusion coefficient reduced by a factor of 2 to 3, this does not prevent the antibiotics from entering into the matrix. However, the antibiotics can be inactivated by binding to the matrix or reacting with the matrix, which inhibit the antibiotics from reaching the depth of the biofilm microcolonies. This diffusion-reaction interaction of the antibiotics is responsible for the prevention of some of the antibiotics from penetrating the biofilm. Though most antibiotics have been shown to have penetration ability in the alginate matrix of the *P. aeruginosa* biofilm, 100% penetration is not achieved in the majority of cases^13^. Thus biofilms show multiple resistance mechanisms such as poor antibiotic penetration, nutrient limitation and slow growth, adaptive stress responses, and formation of persister cells^14, 15^. Additionally, the polysaccharide hydrogel forming the matrix acts as a selective barrier that allows the exchange of nutrients with the surroundings but prevents other substances from entering into it. For example, small, charged molecules are less mobile than large neutral molecules in a biofilm matrix^11^ and this trend has been attributed to the exopolysaccharides of bacterial biofilms^16^. However, not much is known about how the properties of the EPS that allows it to act as a selective diffusion barrier^17^.

Various strategies have been used to eradicate biofilms^18^. Degrading the matrix of the biofilm is an effective strategy for eradicating the biofilm as it increases the antibiotic susceptibility. Macrolide antibiotics have shown to be effective against chronic cystic fibrosis lung disease caused by mucoid *P. aeruginosa* as they play a role in disrupting the matrix^19^. Clarithromycin has been shown to not only kill the bacteria, but also to make the matrix less viscous^19^. In another study, ciprofloxacin and gluteraldehyde have been shown to weaken the material properties of *P. aeruginosa* biofilms^20^. Chemicals such as bismuth dimercaprol caused a reduction in polysaccharide production although it was not effective in killing bacteria^21^.

To understand how effectively the matrix degrades with the use of drugs, several groups have looked at the effect of drugs on the changes in the viscoelastic properties of the matrix. For example, active microrheology studies showed that despite efficient killing of *Escherichia coli* biofilms, the physical properties of the biofilm matrix were preserved^22^. Other studies have found that the mechanical properties of the matrix of *P. aeruginosa* biofilms are unperturbed by most chemical treatments^23^. Among the different chemicals tested, only citric acid affected both elasticity of the matrix and the viability of the bacteria. The ability of antibiotics such as fluoroquinolones to induce multidrug-tolerant persister cells is of serious concern^24^. The persister cells pose a problem when antibiotics are used for treatment as the remnant matrix acts as a scaffold for the persister cells to regrow once the antibiotic pressure is relieved. Hence it becomes necessary to explore the possibility of (i) non-antibiotic drugs (ii) drugs that could diffuse through the matrix without the need of degrading the matrix.

Two weak acid drugs, N-acetyl-L-cysteine (NAC) and acetic acid are emerging as interesting potential therapeutics to treat biofilm-based infections. NAC is an active ingredient in some over-the-counter drug and is commonly used as a mucotytic agent. NAC has been shown to interfere with biofilm formation of Staphylococcus epidermis^25^. Subsequently, NAC has been shown to inhibit the growth of many different bacteria, including *P. aeruginosa* ^26–29^. It is known that a low concentration of acetate with pH corresponding to 6 leads to a bacteriostatic effect, but higher concentration of undissociated acid is needed for cell killing^30^. However, most prior studies relied on optical density measurements and determination of the colony forming units. These methods are reflective of the prevention of the growth of planktonic bacteria shed from the biofilm, rather than the cell growth within the biofilm^31^. Moreover there are no controlled studies on the effect of NAC on intact biofilms and the mechanism accounting for its antibacterial or antibiofilm action is largely unknown^32^.

The antibacterial property of acetic acid has been known for thousands of years^33^. Recently, acetic acid has been used to treat wound infections and is shown to be effective against both planktonic and biofilm states of bacteria. However, only its mechanism of action in killing planktonic bacterial cells has been established^34, 35^. The protonated form of acetic acid has the ability to diffuse through the cell wall of the bacteria leading to the disruption of the cell function by acidifying the cytoplasm. Both acetic acid and NAC exist in the protonated and deprotonated state depending on whether the pH is less than or greater than the pKa. According to chemiosmotic theory, highly lipophilic acids or bases could traverse the cell membrane in both the dissociated and undissociated state, and shuttle protons in a cyclic manner to dissipate the chemical gradient of protons, the membrane potential and total proton-motive force^36^. Undissociated fermentation acids are also lipid-permeable, and experiments with *E. coli* ^37^ and *Lactobacilli* indicated that acetate addition could cause a decline in intracellular pH^38^. The decrease in intracellular pH is explained by an uncoupling model, according to which the deleterious effect due to organic acid is brought about by the acidification of cytoplasm below the permissible pH. Organic acids which act as protonophores increase the inward leak of H^+^ so that H^+^ efflux is not rapid enough to alkalinize the cytoplasm^38^. Fluoride and some organic acids have also been shown to disrupt the respiratory activity of intact cells^39^.

Here we study the action of NAC on mature biofilms and investigate the commonality of NAC with that of acetic acid in order to understand the mechanism of action of NAC. We also study the response of the remnant matrix to changes in solution pH after the bacteria are killed. We show that NAC and acetic acid at pH < pKa can penetrate the matrix of the biofilm and kill 100% of the bacteria embedded in the matrix. After killing the bacteria, the remnant matrix remains intact and behaves as a robust, pH-responsive hydrogel. We also show that the bacteria acts as crosslinkers in the biofilm. Killing the bacteria decreases the crosslinking density, leading to the swelling of the microcolonies, which eventually results in shedding of the dead bacteria from the microcolony.

## Results

### NAC kills biofilm bacteria with 100% efficacy

**Figure 1:**
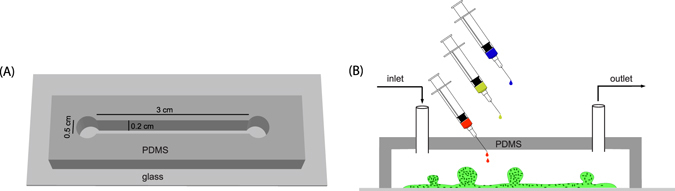
(**A**) Schematic of a flow cell. (**B**) Biofilms are grown in flow cells with a glass base and a PDMS top with a continuous supply of 10% Luria-Bertani broth (LB) supplied using gravity driven flow. The drug is injected into the flow cell through the PDMS while the flow is stopped. Microcolonies grow on the glass base to a size of approximately 200 *μ*m after a period of 2 days.

**Figure 2:**
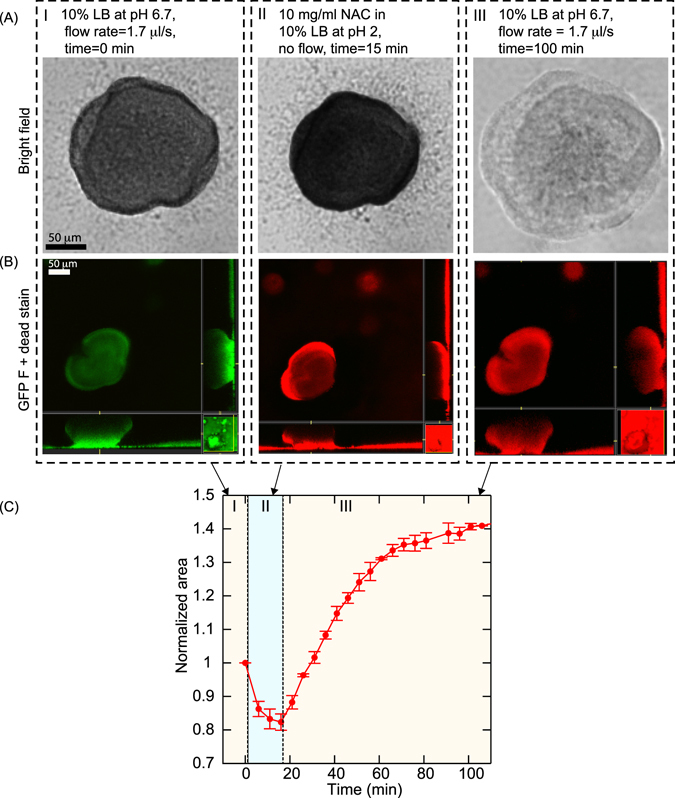
(**A**) Bright field images of a microcolony (I) in 10% LB at pH 6.7 just before treatment, (II) after treating with 10 mg/ml NAC where it shrinks in size, and (III) swells to a size larger than the original size when the pH was switched back to 6.7. Images show the density of the microcolonies also decreased considerably. (**B**) (I) Representative confocal image of microcolonies with bacteria expressing GFP. (II) Green and red channel immediately after the bacteria are treated with NAC. The red fluorescence from the dead stain indicates that the bacteria are dead. (III) Green and red channel after t = 100 min. (**C**) The change in size of microcolonies with time as obtained from the bright field images shows that the microcolonies shrank to 20% of the original size when treated with NAC and swelled to 40% of the original size when the pH was changed back to 6.7. Region I, II, and III correspond to before treatment, during treatment, and after treatment with NAC, respectively. The error bars are the standard deviation of the area obtained from four different microcolonies in the same flow cell.

The mucoid strain of *P. aeruginosa* was grown in a flow cell (Fig. 1) with a continuous flow (flow rate of 1.7 *μ*l/s) of 10% Luria-Bertani broth (LB) at pH 6.7. The bacteria developed mushroom-shaped microcolonies within a period of 2 days. Bright field images of microcolonies are shown in the first panel of Fig. 2A(I). The flow of media was stopped before treating the microcolonies with 1 ml of 10 mg/ml NAC for 15 min. The drug was injected directly into the flow cell, (Fig. 1B) and the quantity of drug used ensures that there is no dilution within the flowcell. We observed that the size of biofilm microcolonies decreased and became darker during treatment, as seen in Fig. 2A(II). After 15 min of treatment, the flow was resumed which flushed out NAC and returned the pH back to 6.7 within 4–5 min. It was observed that the microcolonies gradually increased in size to more than the original size. It should be noted that the microcolonies also became lighter over time as shown in Fig. 2A(III).

Fluorescence images of microcolonies showed that the green fluorescent intensity from the GFP expressing bacteria (Fig. 2B(I)) immediately disappeared upon treatment with NAC as seen in Fig. 2B(II). This could be because the bacteria in the microcolonies were dead. Generally, SYTO9 and propidium iodide (PI) are used to test the viability of bacteria. PI penetrates the disrupted cell membrane and intercalates with the nucleic acids, whereas SYTO9 penetrates both live and dead cells. In *P. aeruginosa* cells, fluorescence signal of SYTO9 from dead bacteria was found to be stronger than for live ones resulting in overestimation of live bacteria^40, 41^. Genetic engineering of bacterial strains with green fluorescent protein (GFP) has been shown to be a useful tool to visualize live bacteria and as an alternative viability marker for bacteria which are impermeable to other dyes^42, 43^. However, the loss of fluorescence is not a certain indication of the viability of the bacteria, as the GFP has been shown to be pH dependent^44^. Hence, we stained the microcolonies with the dead stain‚ propidium iodide, and found that the bacteria in the biofilm stained red indicating that the bacteria in the microcolonies were dead (see Fig. 2B(II and III)). The biofilm microcolonies in the flow cells were left for a period of another 24 hours in a continuous flow of 10% LB and we found that there was no regrowth of the bacteria in the microcolonies indicating a 100% killing of bacteria in the biofilms.

The projected area of the microcolonies were obtained from the bright field images and the variation of the normalized area of the microcolonies as a function of time is shown in Fig. 2C. The area of the microcolony was normalized to have a value of 1 just before drug treatment (region I). The microcolonies shrank by 20% of the original size when treated with NAC (region II) and then swelled to 40% more than the original size when the pH was returned to 6.7 (region III). The data shown in Fig. 2C is the average value from measurements obtained from four different microcolonies in the same experiment. Independent experiments under the same conditions gave reproducible results (Supplementary Figure S4).

Here we make two important observations: (i) NAC diffused into the matrix killing the bacteria embedded in the biofilm within 5 min after treatment, and (ii) the matrix swelled to a size larger than the original size. Next, we will establish that the swelling was due to the bacteria death.

### Swelling of microcolonies has direct correlation with bacteria viability

**Figure 3:**
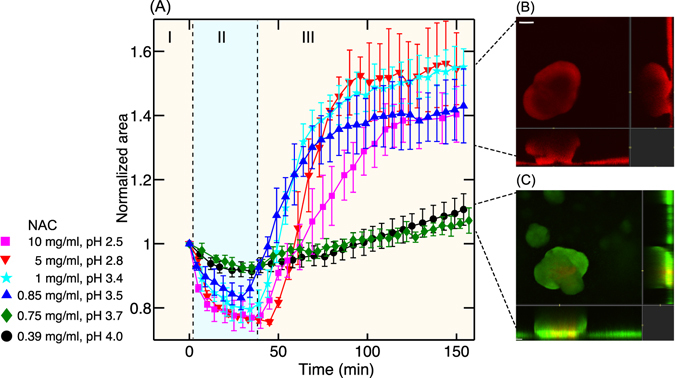
(**A**) Normalized area of microcolonies versus time when treated with different concentrations of NAC. Shrinkage of colonies during treatment (region II) and swelling after treatment (region III) occurs at NAC concentrations greater than 0.85 mg/ml at which the pH < 3.5. For concentrations less than 0.85 mg/ml (pH > 3.5), a gradual increase in size of the microcolonies (region III) indicates the growth of the microcolonies over time due to the presence of live bacteria. (**B**) A representative confocal image of the colonies in which swelling has taken place and (**C**) no swelling (other than due to live bacteria growth) has taken place. The images display green and red channels and correspond to the 100 min time point.

We studied the response of the biofilm as a function of NAC concentration in order to understand if the swelling of microcolonies was related to the killing of bacteria. Biofilm microcolonies were treated with different concentrations of NAC from 10 mg/ml to 0.39 mg/ml. As the pH of NAC depended on its concentration, the pH corresponding to these concentrations ranged from 2.5 to 4. As seen in Fig. 3A, the shrinkage (region II) and swelling (region III) of the microcolonies were observed for concentrations ranging from 10 mg/ml to 0.85 mg/ml of NAC, which corresponds to pH 2.5 to 3.5. The confocal images showed that 100% of the bacteria in the biofilm were killed at concentrations of NAC for which pH < 3.5. Figure 3B is a representative image (green and red channel) of the colonies treated with NAC at pH 2.5 and stained with dead stain. In the next section, we will be showing that the absence of GFP is indicative of cell death.

Only a slight shrinking (<2%) was observed for NAC concentrations < 0.85 mg/ml (pH > 3.5) which is not very significant as compared to 20% shrinking at pH < 3.5. A gradual increase in size of the colonies was observed as shown by the green and the black curves in Fig. 3A when the pH was switched back to 6.7. The confocal images showed that the bacteria were not killed at NAC concentrations < 0.85 mg/ml (pH > 3.5). Figure 3C shows the confocal image (green and red channels) of the biofilm microcolony treated with 0.75 mg/ml of NAC and then stained with dead stain. The green signal is from the GFP of the bacteria and propidium iodide turns the e-DNA at the base of the microcolony red. This type of staining is observed in the control experiments with untreated microcolonies (Supplementary Figure S1A). Hence the gradual increase in size of microcolonies after treatment was due to the growth of the biofilm microcolony.

Taken together, these experiments showed a direct correlation between the swelling of the microcolonies and the viability of bacteria. We also found that pH of the drug is an important factor that determines the killing of bacteria. NAC has a pKa value of 3.3 and the drug penetrated the matrix and killed the bacteria in the biofilm only when the pH < pKa. Hence we conclude that pH relative to pKa is an important factor to facilitate the penetration of the drug into the biofilm. Furthermore, swelling of the microcolonies only occurs when the bacteria were killed in the biofilms.

### Comparison of NAC with acetic acid reveals their commonality

**Figure 4:**
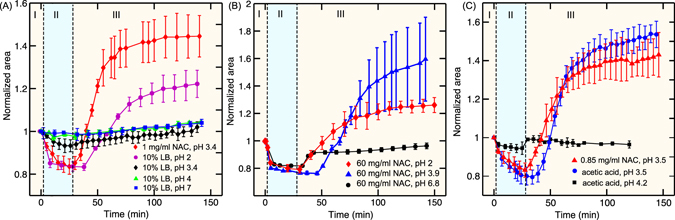
(**A**) Comparison of the effect of HCl and NAC on the killing of biofilm bacteria. The black and red curve compare the behavior of 1 mg/ml NAC (pH 3.4) and 10% LB with pH adjusted to 3.4 using HCl. No swelling at pH 3.4 without NAC indicates no killing of bacteria (confirmed with dead cell staining). (**B**) Effect of NAC with pH adjusted using sodium hydroxide on the killing of biofilm bacteria. The red curve shows the action of 60 mg/ml NAC in 10% LB. The blue and the black curves shows the effect of 60 mg/ml NAC with pH adjusted to 3.9 and 6.8 respectively. (**C**) Comparison of the effect of acetic acid and NAC on the killing of biofilm bacteria. Acetic acid and NAC at pH 3.5 lead to the swelling of the microcolonies. Acetic acid at pH 4.2 and above did not significantly affect the microcolonies.

Although NAC at concentrations corresponding to pH < 3.5 had a bactericidal effect, it was not clear if the killing was due to the low pH or due to the action of NAC. To assess if the bactericidal effect was due to NAC or low pH, we treated the biofilms with 10% LB with pH adjusted to 2, 3.4, 4 and 7 using HCl. Figure 4A shows a comparison of the effect of NAC and pH on the biofilm. The red and the black curves show the response of the microcolony when the biofilms were treated with and without NAC respectively. For the case without NAC, the pH was adjusted using HCl. We found that there was swelling of the colonies only in the presence of NAC. This confirmed that the killing of bacteria at pH 3.4 was due to the action of NAC and not merely a pH effect. These findings were also confirmed using confocal imaging with dead stain (Supplementary Figure S1). It has been reported earlier that HCl at pH 2.5 and less can kill planktonic bacteria^45^. We also observed that 10% LB at pH 2 can penetrate the biofilm matrix and kill the bacteria within (magenta curve in Fig. 4A). These experiments also give a clear indication that the disappearance of GFP when the bacteria are treated with drugs is not a pH effect, but due to the killing of bacteria.

To further confirm that it is the pH of the drug that is important and not just the drug concentration, we treated the biofilm with 60 mg/ml NAC with pH adjusted to 2, 3.9 and 6.8 using sodium hydroxide (Fig. 4B). It can be seen that the microcolonies did not undergo swelling when treated with 60 mg/ml NAC at pH 6.8, indicating that bacteria were not killed (confirmed by dead stain in Supplementary Figure S1). This confirms that NAC can penetrate the biofilm matrix and can have a bactericidal effect only at pH < pKa.

Next, we compared the bactericidal effect of NAC with that of acetic acid (Fig. 4C). Both NAC and acetic acid were diluted with 10% LB to a concentration such that the pH was 3.5. 0.5% acetic acid had a pH of 3.5 in 10% LB. At these conditions, we observed that treatment with both NAC and acetic acid led to the swelling of the microcolony and in both these cases 100% killing of bacteria was observed (Supplementary Figure S1). Hence both NAC and acetic acid can eradicate biofilms at pH < 3.5. This shows a commonality between the acetic acid and NAC. Acetic acid has a pKa value of 4.7 however the same concentration of acetic acid when prepared in 10% LB media had a pH of 3.5. This difference is likely due to the presence of NaCl in the medium which has previously been shown to alter the pH value of acetic acid^46^.

It is known that the protonated form of acetic acid penetrates the bacterial cell wall^34, 47^. Here we also show that the protonated form of both NAC and acetic acid penetrates the biofilm matrix and kills the bacteria within it. Due to the shared properties of acetic acid and NAC, we can postulate the following mechanism of action for NAC in killing bacteria. (i) The protonated state of NAC which predominates at a pH below its pKa (3.3) diffuses through the semipermeable EPS and reaches the bacteria embedded in it. (ii) The protonated state of NAC can then diffuse through the cell wall of the bacteria in the same way as that of acetic acid. Once within the bacteria, NAC deprotonates as the pH is greater than the pKa and hence acidifies the cytoplasm and disrupts the activity of the cell. Hence, the comparison with acetic acid has helped to elucidate the mechanism by which NAC first penetrates the matrix, and then the cell wall which eventually kills the bacteria.

### The remnant EPS behaves like a synthetic polyelectrolyte hydrogel

From all the above observations, it can be seen that the microcolonies swelled to 40% of the original size only when the bacteria are killed. It is known that the changes in pH lead to the shrinking and swelling of the biofilm due to the presence of charges on the matrix. However the phenomenon of excessive swelling (40–60% more than the original size) was intriguing, as one would expect a polyelectrolyte hydrogel to swell to its original size when the pH is switched back to 6.7. In order to test the integrity of the matrix, we studied the response of the matrix when the bacteria are not killed and when they are killed.

**Figure 5:**
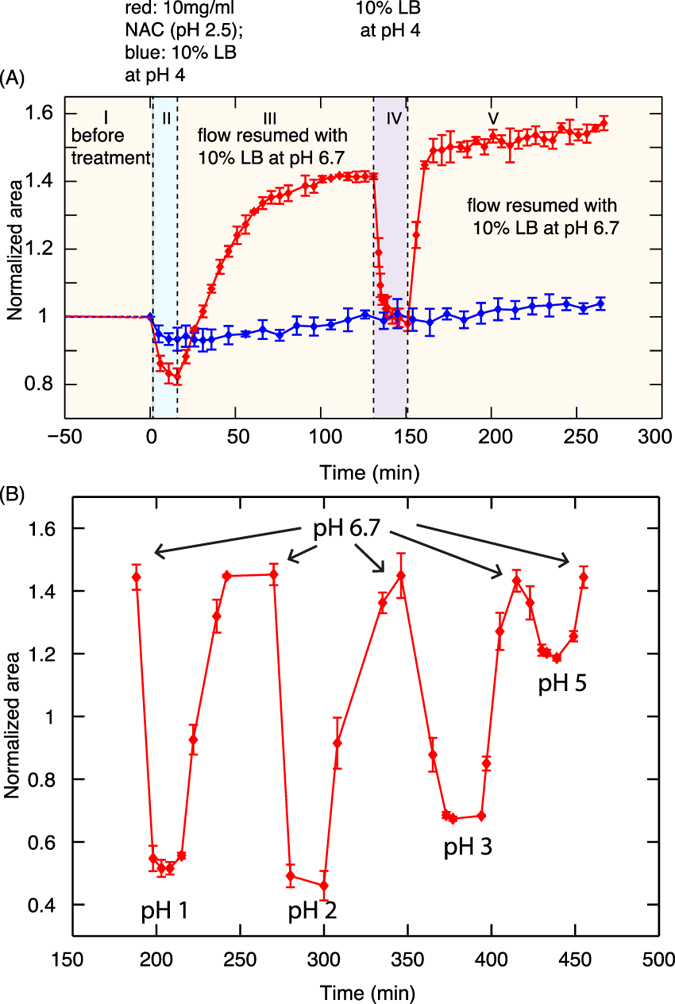
(**A**) The remaining matrix after killing the bacteria behaves differently than when the bacteria are alive. Red curve: When the bacteria are killed by treating with 10 mg/ml NAC (region II), the microcolonies swelled on returning the pH back to 6.7 (region III). The microcolony again shrank on treating with 10% LB at pH 4 (region IV) and swelled with in a shorter time when the pH was switched back to 6.7. Blue curve: The microcolonies did not respond to the changes in pH when the bacteria are not killed in region II. (**B**) The remnant matrix after the bacteria are killed behaved as a pH switchable polyelectrolyte hydrogel - shrinking and swelling reversibly when the pH was switched between a low and high value.

We observed the response of the same set of microcolonies after treating the biofilm with (i) 10% LB with pH adjusted to 4 using HCl and (ii) 10 mg/ml NAC at pH 2.5. At pH 4, the bacteria were not killed and hence there was no expansion as seen in the region (III) of Fig. 5A. The microcolonies did not show any response to pH when the pH was switched again between 4 and 6.7 as shown by region IV and V respectively of Fig. 5A.

When treated with 10 mg/ml NAC at pH 2.5, the bacteria in the microcolonies were immediately killed as indicated by the swelling of the microcolonies to more than the original size (red curve in the region III of Fig. 5A). When the microcolonies were then treated with 10% LB at pH 4, they shrank to almost their original size (region IV), in contrast to what was observed when the bacteria was alive. Hence it is evident that the microcolonies have a different response when the bacteria are alive and when they are dead. It should be noted that immediately after the treatment of microcolonies with NAC, the swelling of the microcolonies in region III followed an exponential increase, with the size increasing at the rate of 4% per min (Supplementary Figure S2). However, in the region IV of Fig. 5A, the size increased at the rate of 18% per min indicating a faster response to the change in pH when the bacteria were killed (Supplementary Figure S2). To further probe the properties of the matrix, we killed the bacteria and then treated the matrix with 10% LB with pH adjusted to 1, 2, 3 and 5, each time alternating with pH 6.7. We found that the matrix was robust and pH switchable as shown in Fig. 5B. Thus we demonstrate that after the bacteria is killed, the matrix behaved as a robust, pH responsive polyelectrolyte hydrogel.

Hence we find that the excessive swelling of the microcolonies when the bacteria are killed is not due to degradation of the matrix, rather the matrix remains intact and maintains the mushroom-shaped structure of the microcolonies. We hypothesize that the excessive swelling of the microcolonies is the result of the decrease in the crosslinking density of the biofilm matrix.

### Excessive swelling of microcolonies is due to the shedding of dead bacteria

**Figure 6:**
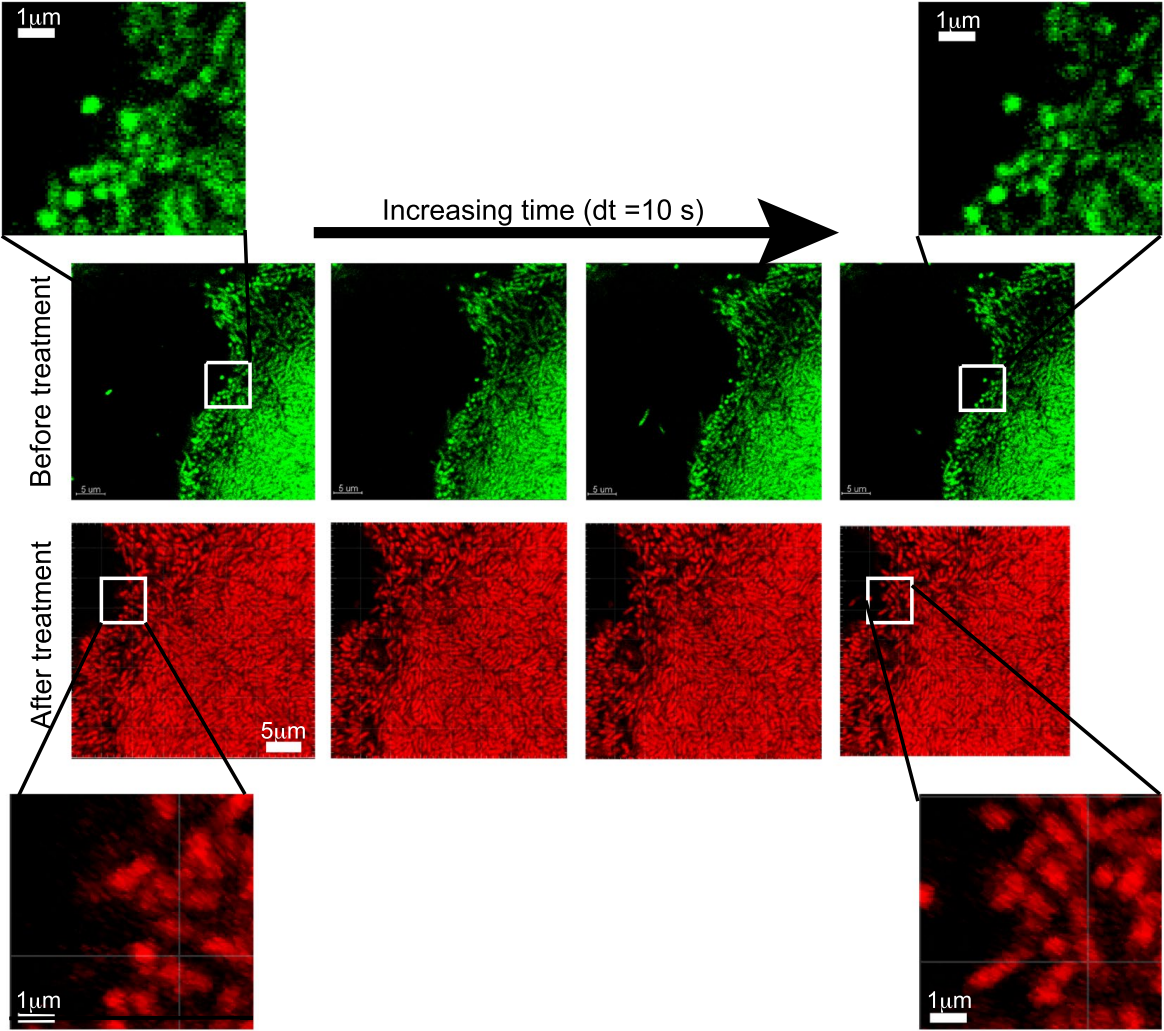
Confocal images of the microcolony before and after treatment with 10 mg/ml NAC for 15 min. The images are snapshots at 10 s time intervals. (**A**) When the bacteria are alive there is no relative motion of the individual bacteria within this time span. (**B**) When the bacteria are killed, there is a rearrangement in the bacterial cells at the edge of the colony within a period of 40 s. Shedding of the bacteria is also shown in Supplementary Movie 1.

To understand the cause of excessive swelling of the microcolonies followed by the killing of the biofilm bacteria, confocal images of the edge of a microcolony were obtained and the behaviour of the bacterial cells attached to the matrix before and after treatment were observed. Figure 6 shows snapshots of the edge of the same microcolony taken every 10 s before and after treatment with NAC. Qualitatively, it can be seen that the relative position of the live bacteria (top panel in Fig. 6) does not change during this time span, but the relative position of the dead bacteria changes (lower panel of Fig. 6) and over time, the dead bacteria diffuses out of the microcolony (Supplementary Movie 1). The bacteria can diffuse out of the microcolony only if the interaction between the bacteria and the matrix is broken.

## Discussion

Extensive use of antibiotics has led to the increased tolerance and resistance of bacterial biofilms against these drugs and hence necessitating the development of novel strategies to control biofilm-based infections^31^. The diffusion of antibiotics through biofilms has been assessed by concentration measurements and visualization of bactericidal effects in the depths of *in vitro* biofilms^48^. The impaired penetration of antibiotics into the biofilm has been hypothesised to be due to the binding of the positively charged drugs to the negatively charged matrix, or to the deactivation of the antibiotics on the surface faster than they can diffuse. This retardation may allow more time for bacteria to implement adaptive stress responses^48^. Aminoglycoside penetration into a *P. aeruginosa* biofilm was significantly hindered by binding to the extracellular alginate but markedly improved after the addition of alginate lyase^49^. The biofilm matrix is generally found to be robust and unaffected by most chemicals or drugs^23^. Hence it is important to change the treatment strategies and find new methods/drugs that would penetrate the matrix quickly and prevent the development of superbugs. Here we explored a new class of weak acid drugs and studied the conditions under which these drugs can effectively penetrate the matrix and kill the bacteria within the biofilm with 100% efficacy.

The use of NAC has been proposed as an alternative pharmacological approach to control bacterial biofilm growth in human diseases. *In vitro* studies reported that NAC decreases biofilms formed by a variety of bacteria^29, 25, 28, 50^. It is also shown that biofilms grown in the presence of NAC showed reduced production of extracellular polysaccharide while promoting the disruption of mature biofilm^50^. These studies used the method of growing bacteria in the presence of NAC for about 24 hours and determining the number of colony forming units. These studies are not reflective of the ability of NAC to diffuse into the biofilm matrix and eradication of the biofilm bacteria, nor do they provide information on the time taken for the activity of NAC. There are neither controlled studies to determine the conditions under which NAC is effective in eradicating biofilm, nor elucidation of the mechanism of action of NAC.

Here we used a microfluidic platform to study the response of biofilms to weak acid drugs. This set up allowed us to observe the response of the same microcolony over a long period of time while the drug/ pH conditions in the fluidic cell are being changed. The bacteria used in this study, mucoid strain of *P. aeruginosa*, has the ability to produce excess alginate together with other polysaccharides like Psl and Pel and develop into a distinct mushroom-shaped microcolonies ranging in size from about 100–200 *μ*m in size. The Young’s modulus of the matrix increases with colony size, and the microcolonies expresses larger amount of EPS than the plains of the biofilm^6, 7^. We have also looked at the 3 d old biofilms and the results were the same as those obtained from 2 d old biofilm. This provides a model system to discern if the drug penetrated into the matrix or acted on the periphery.

We found that NAC has a bactericidal effect and has the ability to penetrate the full depth of the microcolonies with in 7 min and kill 100% of the bacteria (Supplementary Figure S3). The pH of NAC compared to its pKa is the key factor that facilitated the drug in (i) entering the biofilm matrix and (ii) killing the bacteria within the biofilm by diffusing through the cell wall. According to Henderson-Hasselbalch equation, at pH = pKa, 50% of the weak acid is ionized and at pH < pKa and pH > pKa there is a logarithmic decrease and increase in ionization respectively. It has been reported that acetic acid in the protonated form has the ability to penetrate the cell wall of the bacteria^34, 35, 51^. Physicochemical studies have shown that NAC has a -COOH group (pKa = 3.3) and -SH group (pKa = 9.87) because of which NAC predominates as NACH_2_ at pH < 3.3, as NACH^−^ at pH between 3.3 and 9.87 and as NAC^2−^ at pH > 9.87^52, 53^. Pharmacological studies have reported that neutral form of NAC that predominates at pH < 3.3 allowed membrane penetration from gastric fluid (pH 1.5–3.3) by passive diffusion^53^. However the role of NAC in killing bacteria has not been reported. From our experiments comparing the action of acetic acid and NAC on biofilm, we postulate that the mechanism of action of NAC in penetrating the matrix and the cell wall is the same as that of acetic acid - the neutral form of NAC diffuses into the bacterial cell. As the pH inside the bacteria is high (around 7.6), NAC dissociates and acidifies the cytoplasm, hence denaturing proteins and causing DNA damage.

The EPS acts as a selective barrier that controls the exchange of nutrients with the surroundings but preventing other substances from entering into it and this has been attributed to the exopolysaccarides of bacterial biofilm^16^. Our experiments showed that NAC and acetic acid penetrated the matrix when the pH < pKa. Hence we propose that NAC or acetic acid in the undissociated form (pH < pKa) can penetrate the matrix and eventually reach the bacterial cell embedded deep within the matrix. It should be noted that the experiments reported so far are on the mucoid strain of *P. aeruginosa*. The major component of the matrix, the alginate, along with other polysaccharides like Pel and Psl and the entanglements are responsible for the cohesiveness of the matrix in this strain of bacteria^54^. It has been shown that acetic acid prevented planktonic growth and eradicated biofilm formed by a range of clinically isolated bacterial strains^51^. Moreover, here we have shown that the ability of the drug to penetrate the biofilm matrix and the bacterial cell depends on the pKa of the drug relative to the pH of the solution. This relation of drug efficacy to the solution-pH/drug-pKa is general and should be tested to see if it will apply to other bacterial strains. A general outcome from our work is that drug pKa should be considered when considering drug design and delivery vehicles for biofilms.

Another important outcome of this study is the role of bacteria in maintaining the structure of the biofilm matrix and the response of the matrix when the bacteria are dead. Studies on artificial biofilms have shown that formation of biofilms involve the physical interaction between the bacteria and the matrix and the viscoelastic properties of the biofilm are due to the self assembly of the bacteria and the matrix polymer^55^. Inspired by this work, we proposed the following mechanism to explain the swelling of the remnant matrix.

**Figure 7:**
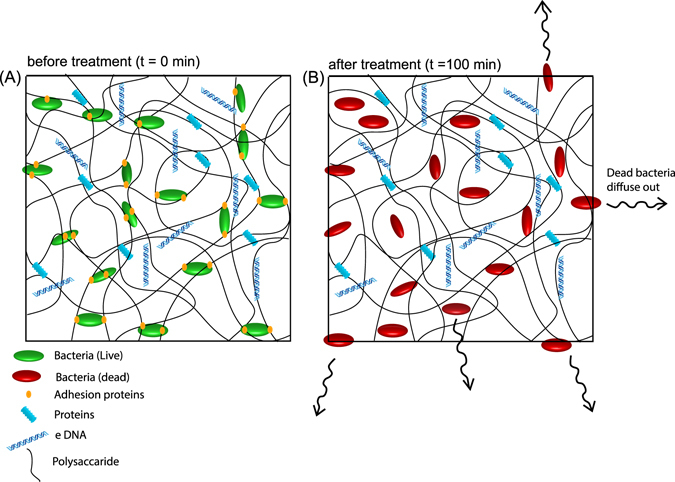
Model of bacterial cells embedded in the matrix comprising of polysaccharides, proteins, and DNA. (**A**) When the bacteria are alive, they act as crosslinks due to the surface adhesive proteins holding the matrix together. (**B**) When the bacteria are dead, the crosslinks are broken due to the denaturation of the surface adhesive proteins, and this leads to the swelling of the microcolonies, which eventually leads to dead bacteria passively diffusing out of the colonies.

Bacterial-cell surface-associated proteins have been known to bind to the polysaccharide matrix to maintain the structural integrity of the biofilm^5^. Proteomic studies have shown that 30% of the proteins identified in the matrix of *P. aeruginosa* are outer membrane proteins and are abundant in the biofilm than in the planktonic state of bacteria indicating that the surface proteins play an important role in biofilm formation^56^. Though the role of majority of the proteins have not be identified, it has been shown that one of the proteins links the cell to the Psl polysaccharide^57^. Hence, the live bacteria interacts with the matrix components by the surface proteins and can act as crosslinkers holding the matrix together as illustrated in Fig. 7A. Killing of bacteria leads to the degradation of the surface proteins and thus breaking of the crosslinks. The microcolonies then have decreased crosslinking density, which leads to its swelling beyond the original size and the shedding of the loose, dead bacterial cells (Fig. 7B). This observed swelling is analogous to what happens in a synthetic polymer gel when the crosslink density decreases due to hydrolysis or other degradation mechanisms. The decrease in biomass can be observed from the less dense microcolonies after treatment with NAC (Fig. 2AIII). It should also be noted that the size of the microcolony in Fig. 5A, region V, gradually increased over time indicating that the bacterial cells continue diffusing out of the colony with time (Supplementary Movie 1), due to a gradient in concentration of bacterial cells inside and outside the EPS.

The shedding of the dead bacterial cells leaves behind a robust matrix which reversibly swells and deswells at high and low pH respectively. The decrease in bacterial crosslinks, however, does not disintegrate the matrix as the majority of the crosslinks are due to the proteins or electrostatic interactions. We found that when the microcolonies were treated with proteinase K, the colonies immediately disintegrated (Supplementary Figure S5). Studies have shown that growing bacteria in the presence of NAC showed reduced production of EPS^50^, however, there are no studies which looked at the response of a pre-formed biofilm EPS of a mucoid strain of *P. aeruginosa* to NAC. We have shown that the remnant EPS after the bacteria are killed behaves similar to a synthetic polyelectrolyte hydrogel. The remnant matrix is pH switchable and a robust soft matter material. This pH responsive behaviour is similar to the response observed in sodium alginate hydrogels^58^ and hence may serve as an alternative alginate hydrogel source for some applications. More broadly, the ability to both kill and remove bacteria from the EPS offers a new route to produce bacteria-free EPS for applications ranging from cosmetics, biopharma, and waste water treatment^59–61^.

## Conclusions

Here we used microfluidic platform to study the efficacy of weak organic acid drug, N-acetyl-L-cysteine (NAC), on eradication of biofilm bacteria and investigated the commonality of this drug with that of acetic acid on mucoid strain of *Pseudomonas aeruginosa*. We showed that at pH < pKa, NAC and acetic acid have the ability to penetrated the matrix barrier and then diffuse into the bacterial cell killing 100% of the bacteria in the biofilm. We showed that live bacteria acts as crosslinkers in the biofilm and killing the bacteria decreases the crosslinking density, leading to the swelling of the microcolonies which eventually results in shedding of the dead bacteria from the microcolony. The remnant matrix is a robust, pH-responsive soft matter material.

## Methods

### Flow chamber

Poly(dimethylsiloxane) (PDMS) flow cells were fabricated from a 3D printed stamp, using a Sylgard 184 kit (Dow Corning, UK). The flow cell had a straight channel with dimensions 0.2 cm × 0.5 cm × 3 cm (height × width × length) (Fig. 1A). PDMS monomer and the curing agent were mixed in a 10:1 ratio (w/w) and this mixture was placed in a vacuum chamber for 1 h to remove air bubbles trapped during mixing. The mixture was then slowly poured into the mold and left at room temperature for 24 h after which it was incubated at 70 °C for 1 h. Once the PDMS cooled, it was removed from the mold, oxygen plasma treated and bonded to a glass coverslip. Intet and outlet holes of 1 mm diameter were created using Uni-CoreTM punchers (Sigma-Aldrich, St. Louis, MO, USA) before bonding.

### Flow system

Continuous flow of nutrients was provided by a simple, gravity fed system, which comprised of an inverted conical flask (1 L), with a one-hole rubber stopper. A clinical transfusion kit (Baxter, UK) was inserted into the rubber stopper and this allows for adjustment of the flow rate. This system provides a continuous flow rather than a pulsating flow as is in the case when using a peristaltic pump.

### Biofilms formation

The alginate overproducing *Pseudomonas aeruginosa mucA* strain^62^ was used in all experiments. Fluorescently-tagged strains were constructed by the insertion of a mini-Tn7-enhanced green fluorescent protein (eGFP)- Gm^*r*^ cassette as described^63^. Overnight cultures of *P aeruginosa* strains were grown in Luria-Bertani broth (5 g/L NaCl, 5 g/L yeast extract, 10 g/L tryptone) at 37 °C under shaking conditions (200 rpm). The overnight *P aeruginosa* culture was diluted to an optical density at 600 nm (OD_600_) of 0.4, and 350 *μ*L was injected into the flow cell and incubated for 1 h for the bacteria to attach to the glass surface. After the initial attachment, 10% LB medium was supplied to the biofilm at a flow rate of 1.7 *μ*l/s. The biofilms were then allowed to grow and mature for a period of 2 d.

### Staining and imaging

Propidium iodide (PI) (Thermo Fisher Scientific, USA) at a concentration of 3 *μ*l/mL was used to stain the dead bacterial cells of the biofilm for 20 min. PI is an intercalating agent that stains the nucleic acid of dead cells and is used to differentiate dead and live cells.Three dimensional image stacks of biofilm colonies of different sizes were acquired using FluoView 1000 confocal microscope (Olympus Japan) with a 20x or 60x oil immersion objective. Two image channels were acquired for each stack, GFP 488 and Alexa 594. The number of z-stacks depended on the height of the colonies. Time lapse image of shrinkage and expansion of the biofilm microcolonies were obtained by the an inverted Olympus IX71 fluorescence microscope with a 10x long working distance objective.

## Electronic supplementary material

Supplementary Information

Supplementary Movie 1
