# Supplementary material for: Mechanistic action of weak acid drugs on biofilms
Source: Sci Rep. 2017 Jul 6;7:4783. doi: 10.1038/s41598-017-05178-3 (PMC5500524; doi:10.1038/s41598-017-05178-3)
Supplement: Supplementary file 1 — Supplementary Information [file 41598_2017_5178_MOESM1_ESM.pdf]

Mechanistic action of weak acid drugs on biofilms

Binu Kundukad[1], Megan Schussman[2], Yang Kaiyuan[3], Thomas Seviour[4], Yang Liang[4,5], Scott A. Rice[4,5], Staffan Kjelleberg[4,5,6], and Patrick S. Doyle[1,7,*]

[1]BioSystems and Micromechanics (BioSyM) IRG, Singapore MIT Alliance for Research and Technology (SMART), Singapore

[2]Department of Brain and Cognitive Science, Massachusetts Institute of Technology, Cambridge, Massachusetts 02139, USA

[3]Department of Pharmacy, National University of Singapore, Singapore

[4]Singapore Centre for Environmental Life Sciences Engineering, Nanyang Technological University, Singapore

[5]School of Biological Sciences, Nanyang Technological University, Singapore

[6]Centre for Marine Bio-Innovation and School of Biotechnology and Biomolecular Science, University of New South Wales, Sydney, NSW, Australia

[7]Department of Chemical Engineering, Massachusetts Institute of Technology, Cambridge, Massachusetts 02139, USA

Supplementary Information

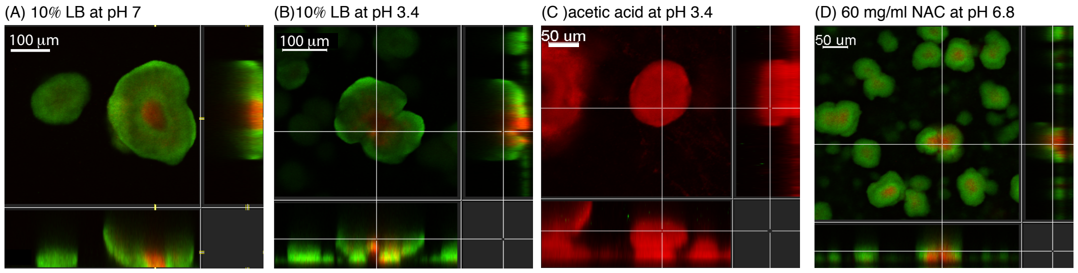

Supplementary Figure S1: Microcolonies without NAC were not killed at low pH. (A) Microcolonies in 10% LB at pH 7 when treated with dead stain showed a red color at the base of the stack due to the staining of e-DNA.

(B) Microcolonies treated with 10% LB at pH 3.4 did not kill the bacteria.

(C) Microcolonies treated with acetic acid at pH 3.4 killed the bacteria within the biofilm.

(D) Microcolonies treated with 60 mg/ml NAC with pH adjusted to 6.8.

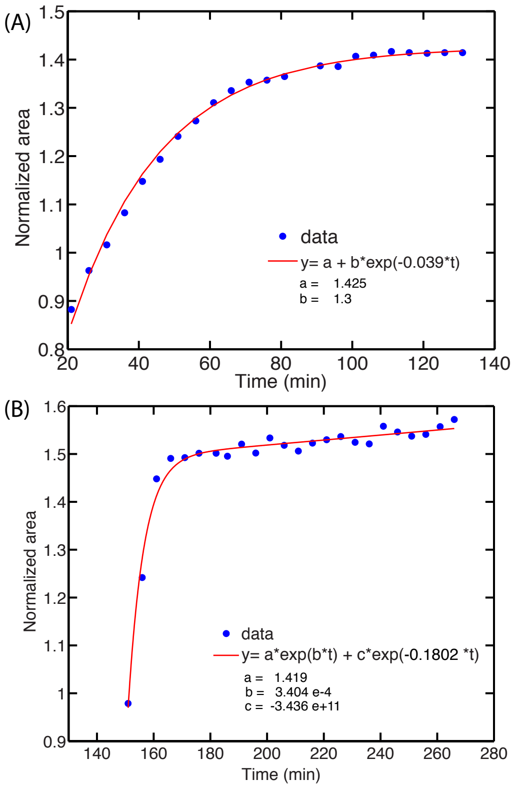

Supplementary Figure S2: (A) The swelling of the microcolonies after the treatment with 10 mg/ml NAC and restoring the pH to 7, follows an exponential increase in size at the rate of 4% per min. The slow swelling is due the breaking of crosslinks and the bacteria diffusing out during the swelling process. (B) The remnant matrix which was initially deswelled on treating with pH 4, swelled on returning the pH to 7 at the rate of 18% per min. The fast response is due to the remnant matrix behaving as a synthetic hydrogel. The matrix continues to swell gradually after t=180s, as more bacteria diffuse out with time. The two term exponential here accounts for the gradual increase in the size after the initial fast response.

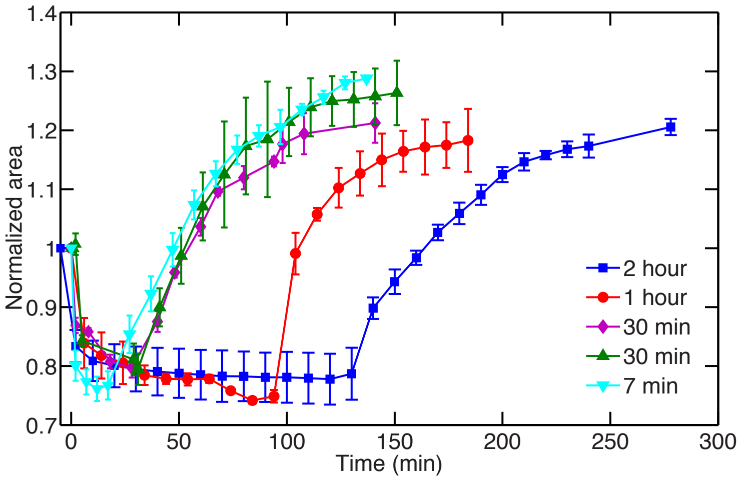

Supplementary Figure S3: Response of the microcolonies as a function of incubation time of NAC. Microcolonies are treated with 10 mg/ml NAC for different incubation times. Incubation times as short as 7 min led to 100% killing of bacteria, which resulted in swelling of the microcolonies.

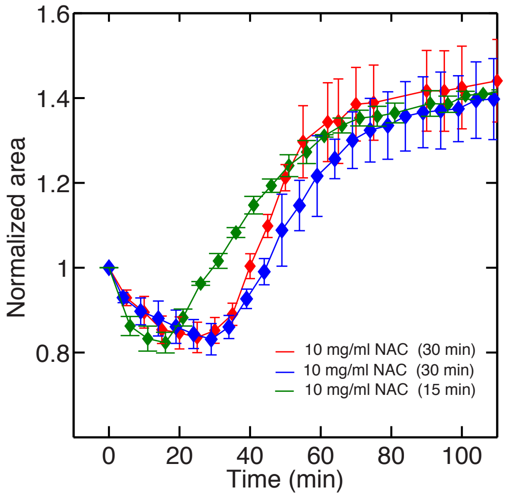

Supplementary Figure S4: Independent experiments in which biofilm microcolonies were treated with 10 mg/ml of NAC for 30 min (red and blue curve) and with 10 mg/ml of NAC for 15 min (green curve).

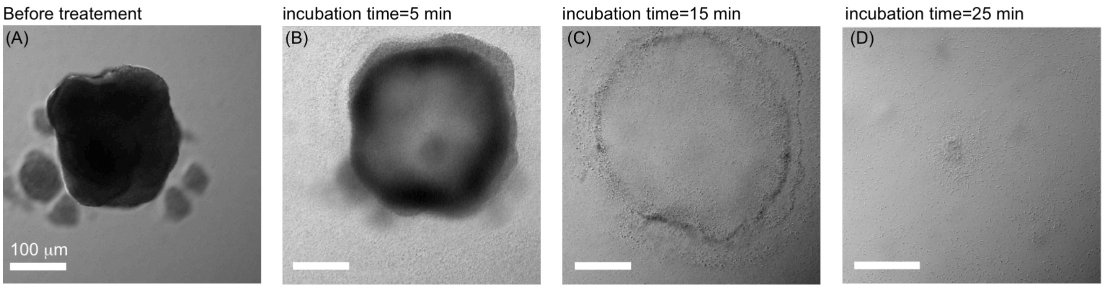

Supplementary Figure S5: Bright field images of the microcolonies (A) before treatment, and during incubation with proteinase K after (B) 5 min (C) 15 min and (D) 25 min.

Supplementary Movie 1: Microcolonies treated with 10 mg/ml NAC led to the killing of the bacteria as seen by the red color from the dead stain. Dead bacteria diffuse out of the microcolony when the microcolonies start swelling on returning the pH to 7. The direction of flow is to the left.
